# Efficacy of Clindamycin Vaginal Ovule (3-Day Treatment) vs. Clindamycin Vaginal Cream (7-Day Treatment) in Bacterial Vaginosis

**DOI:** 10.1155/S1064744901000035

**Published:** 2001

**Authors:** Jack Sobel, Jeffrey F. Peipert, James A. McGregor, Charles Livengood, Maureen Martin, Jill Robbins, Charles P. Wajszczuk

**Affiliations:** 1 Division of Infectious Diseases Detroit Medical Center Wayne State University School of Medicine 3990 John R. Street Detroit MI 48201 USA; 2 Women and Infants Hospital Brown University School of Medicine Providence RI USA; 3 Denver Health Medical Center Denver CO USA; 4 Duke University Medical Center Durham NC USA; 5 Pharmacia and Upjohn Kalamazoo MI USA

## Abstract

*Objective:* To compare the efficacy and safety of a 3-day regimen of clindamycin vaginal ovules with a 7-day regimen of clindamycin vaginal cream for the treatment of bacterial vaginosis (BV)

*Methods:* Women with a clinical diagnosis of BV were treated with a 3-day course of clindamycin ovules or a 7-day course of clindamycin cream administered intravaginally. Three hundred and eighty-four patients received study drug and were included in the evaluable patient population (ovule group, *n* = 204; cream group, *n* = 180). Assessments included pelvic examination and diagnostic testing. Primary efficacy endpoints were a resolution of two of three diagnostic criteria at the first follow-up visit and three of three diagnostic criteria at the second.

*Results:* Cure rates in the evaluable patient population were similar between treatment groups: 53.7% (109/204) for the ovule group and 47.8% (85/180) for the cream group (*p* = 0.2471, 95% CI– 4.1–16.0%). The most commonly reported medical event, vulvovaginal pruritus, had similar incidence in both treatment groups.

*Conclusions:* A 3-day course of clindamycin vaginal ovules is as effective and well-tolerated as a 7-day course of clindamycin vaginal cream in the treatment of BV.


### Efficacy of clindamycin vaginal ovule (3-day treatment) vs. clindamycin vaginal cream (7-day treatment) in bacterial vaginosis

**Jack Sobel[1], Jeffrey F. Peipert[2], James A. McGregor[3], Charles Livengood[4], Maureen Martin[5], Jill Robbins[5] and Charles P. Wajszczuk[5]**

[1]*Division of Infectious Diseases, Detroit Medical Center, Detroit, MI*
[2]*Women and Infants Hospital, Brown University School of Medicine, Providence, RI*
[3]*Denver Health Medical Center, Denver, CO*
[4]*Duke University Medical Center, Durham, NC*
[5]*Pharmacia and Upjohn, Kalamazoo, MI*

***Objective:*** To compare the efficacy and safety of a 3-day regimen of clindamycin vaginal ovules with a 7-day regimen of clindamycin vaginal cream for the treatment of bacterial vaginosis (BV).
***Methods:*** Women with a clinical diagnosis of BV were treated with a 3-day course of clindamycin ovules or a 7-day course of clindamycin cream administered intravaginally. Three hundred and eighty-four patients received study drug and were included in the evaluable patient population (ovule group, $n$ = 204; cream group, $n$ = 180). Assessments included pelvic examination and diagnostic testing. Primary efficacy endpoints were a resolution of two of three diagnostic criteria at the first follow-up visit and three of three diagnostic criteria at the second.
***Results:*** Cure rates in the evaluable patient population were similar between treatment groups: 53.7% (109/204) for the ovule group and 47.8% (85/180) for the cream group ($p$ = 0.2471, 95% CI –4.1–16.0%). The most commonly reported medical event, vulvovaginal pruritus, had similar incidence in both treatment groups.
***Conclusions:*** A 3-day course of clindamycin vaginal ovules is as effective and well-tolerated as a 7-day course of clindamycin vaginal cream in the treatment of BV.

Key words: CLINDAMYCIN; INTRAVAGINAL THERAPIES; VAGINAL INFECTION

Bacterial vaginosis (BV) is a commonly reported vaginal infection among women of child-bearing age. Among pregnant women, BV has an incidence of 15% to 20%[1] and has been associated with an increased risk of pregnancy complications, including preterm labor, low birth weight, chorioamnionitis, pelvic inflammatory disease, and postpartum endometritis[2–5]. In nonpregnant women, untreated asymptomatic BV is associated with complications following gynecologic procedures, in addition to presenting as a symptomatic infection[1,2,6,7].

Systemic or local antibiotic therapy remains the standard regimen for the treatment of BV. The most widely recommended therapy is oral metronidazole (2 g single dose or 500 mg twice daily for 7 days)[6,8]. Its use, however, is constrained by the potential for systemic adverse effects; thus, many

Grant sponsor: Pharmacia and Upjohn, Bridgewater, NJ.
Correspondence to: Jack Sobel, MD, Division of Infectious Diseases, Detroit Medical Center, Wayne State University School of Medicine, 3990 John R. Street, Detroit, MI 48201, USA

clinicians may prefer local antibiotic therapy for the routine treatment of BV[9]. Currently used intravaginal therapies are clindamycin vaginal cream (2%, once daily for 3 or 7 days) and metronidazole gel (0.75%, once or twice daily for 5 days). Several studies that have compared the oral and local therapeutic regimens have found similar efficacy and safety profiles when clindamycin is used in the treatment of BV[6,10–13]. In 1993, it was demonstrated that a 3-day course of clindamycin vaginal cream was as effective as the standard 7-day regimen[14]. Subsequent studies have confirmed the efficacy of this regimen in the treatment of BV[15,16]. Since then, the 3-day course has been approved for the treatment of uncomplicated BV in several European countries and recently (March, 1998) in the United States. Interest in both a shorter therapeutic regimen and an alternative vaginal-dosage form led to trials with the clindamycin vaginal ovule. In this study, efficacy and safety of a 3-day clindamycin vaginal ovule regimen are compared to those of the standard 7-day clindamycin vaginal cream regimen for the treatment of BV.

### SUBJECTS AND METHODS

Prior to initiation, the study was reviewed and approved by independent review committees responsible for ensuring the rights and safety of the research subjects, in accordance with the Declaration of Helsinki and the National and International Good Clinical Practice Guidelines and Ethical Standards, including those of the countries in which the study was conducted. It was conducted in accordance with the ethical principles that have their rights and origins in the Declaration of Helsinki. Signed, written, informed consent was required for all patients.

Patients to be included in this multicenter, prospective, randomized, observer-blinded study were symptomatic women aged 16–60 years having a clinical diagnosis of BV with fulfillment of all the following Amsel criteria: (1) vaginal fluid pH > 4.5, (2) 'fishy' amine odor upon addition of potassium hydroxide (KOH) to the vaginal fluid, and (3) presence of clue cells in the vaginal fluid upon microscopic examination. These criteria were selected based on their widely accepted reliability as diagnostic indicators of BV[17]. In addition, all subjects were required to have a Gram stain score compatible with BV based on the 0–10 scoring system and colleagues of Nugent and colleagues[18]. Criteria for exclusion from the study were as follows: allergy to clindamycin or lincomycin, pregnancy or breast-feeding; systemic or vaginal antimicrobial therapy within the previous 2 weeks; previous enrollment in the study, necessity of using nonprotocol antibiotics; positive cultures or tests for *Neisseria gonorrhoeae*, *Candida albicans*, *Trichomonas vaginalis* or *Chlamydia trachomatis*; atrophic vaginitis; clinical evidence of herpes virus infection; and anticipation of menses during treatment or follow-up visits. Patients were also excluded when they had any other condition or disease that, in the investigator's opinion, should exclude the patient from the study.

Treatment regimens were either clindamycin vaginal ovule, 100 mg, digitally inserted intravaginally at bedtime for 3 consecutive days, or clindamycin vaginal cream 2%, 5 g (1 applicator equivalent to 100 mg clindamycin), applied intravaginally at bedtime for 7 consecutive days. The 100-mg dose of clindamycin ovule was selected based on its milligram equivalence to one applicator of clindamycin cream. Patients were randomly assigned to receive one of the drug regimens based on a random list of patient numbers at a 1:1 ratio.

Following informed consent, pretreatment evaluations included history and pelvic examination; description of vaginal discharge; pH of vaginal fluid; smear of vaginal fluid for clue cells; test for fishy odor after addition of KOH; Gram stain score of vaginal fluid; and appropriate diagnostic testing for *T. vaginalis*, *C. albicans*, *C. trachomatis* and *N. gonorrhoeae*. Follow-up examinations (12–16 days and 18–52 days after treatment) included repeat diagnostic tests for pH, clue cells and odor; repeat Gram stain; description of vaginal discharge; vulvovaginal examination; tests for *C. albicans* and *T. vaginalis* if symptomatic; treatment compliance information (first follow-up only); and assessment of medical events and concomitant medications.

Overall patient outcome (cure or failure) was determined by three criteria (amine odor, pH and clue cells). Cure was defined as a resolution of two of three diagnostic criteria at the first follow-up visit and three of three criteria at the second visit. Secondary efficacy measures included patient evaluation of efficacy and Gram stain score. Patient evaluations of efficacy, using the terms ‘cured’, ‘improved’ or ‘failed’ were completed at the second follow-up visit. Gram stain outcomes for vaginal fluid smears, obtained at both first and second follow-up visits, were evaluated for BV using a standardized morphotype scoring system[18].

Patients in the study were considered nonevaluable if any of the following applied: failure to meet selection criteria, less than 3 days of treatment with ovules (or lapses in ovule dosing), less than 6 days or more than 8 days of treatment with cream (with an allowable lapse of 1 day), menses during protocol therapy or follow-up visit, nonprotocol systemic or vaginal antimicrobial therapy during protocol therapy or before a follow-up visit (with the exception of patients deemed to be treatment or side-effect failures), failure to return for follow-up visit (except for clinical or side-effect failures), douching during protocol therapy or within 2 days of a follow-up visit, development of a symptomatic or concomitant genital infection (i.e. yeast vaginitis or trichomoniasis), or for any other reason that in the opinion of the investigator and monitor made the patient nonevaluable.

All patients enrolled in the study were included in the safety analysis unless no protocol medication was taken. Safety was evaluated by patient-reported medical events. The reporting period for medical events began after the first dose of study medication and ended at the final follow-up visit. Additional events reported were those occurring outside the medical events-reporting period judged by the investigator as possibly related to the study medication. When study patients became pregnant while receiving study medication or within 30 days of discontinuation, appropriate measures were taken to follow the pregnancy until its completion, and results were reported for exposure *in utero*.

Data quality-control measures were implemented to ensure the reliability of the data collected, including periodic visits to study sites by the sponsor to ensure proper adherence to study protocol and routine site audits to ensure accuracy and consistency of data collected.

All statistical tests were two-sided tests; $p$ values $\leq 0.05$ were deemed statistically significant. Differences between treatment groups for efficacy endpoints were analyzed using either Pearson $\chi^2$ tests or Cochran–Mantel–Haenszel tests. Other categorical variables (race, medical history, pretreatment pelvic examination abnormalities and proportion of patients reporting medical events) were analyzed using Fisher’s exact test. Continuous variables (age, weight and previous episodes of BV) were analyzed using one-way analysis of variance. Categorical variables (other than medical events) were analyzed using Pearson $\chi^2$ tests (with the exclusion of the nonassessable variable for the primary efficacy measure). The proportion of patients reporting at least one medical event and the proportion of patients reporting at least one drug-related medical event were analyzed using Fisher’s exact test. Based on the probability of correctly concluding that the rate of success for the clindamycin vaginal ovule was not more than 15% less than the expected 65% success rate for the cream, the power was 0.81.

### RESULTS

Of the intent to treat (ITT) patients, 384 patients were considered evaluable, 204 in the ovule group and 180 in the cream group. The main reasons for nonevaluability are summarized in Figure 1. The most common reasons for nonevaluability were failure to meet inclusion criteria postenrollment and noncompliance with the dosing regimen. The percentage of patients considered nonevaluable for each primary reason was similar between treatment groups.

Table 1 summarizes demographic data for patients in the ITT population. There were no statistically significant differences in patient demographics (age, weight and race) for ITT patients between treatment groups. The demographic profile for the evaluable patient population was similar to that of the ITT population (data not shown).

No statistically significant differences were noted between treatment groups in either the ITT

or the evaluable patient population with respect to present or past medical history. The number of previous episodes of BV, as well as the incidence of abnormal findings in the pelvic examination, was also similar between treatment groups.

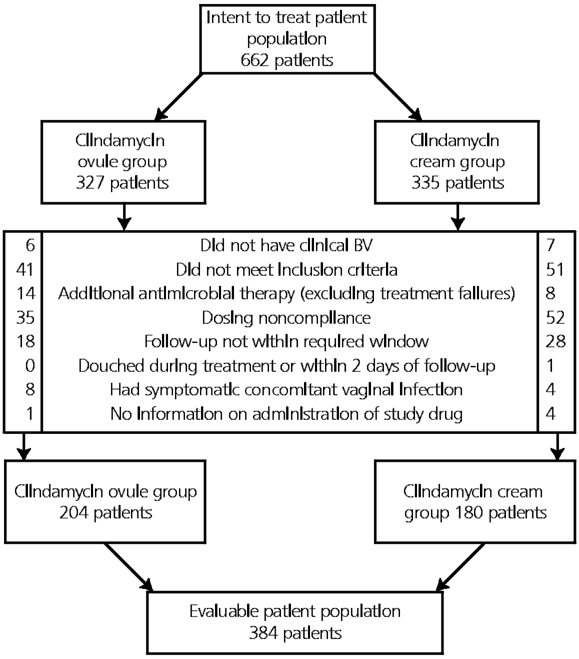

**Figure 1** Algorithm of patient selection. BV, bacterial vaginosis

### Fourteen-day assessment

Based on three diagnostic criteria (clue cells, pH and amine odor), 86.2% of patients in the ovule group (175/204) were cured compared to 75.4% of patients in the cream group (135/180), reflecting statistical significance ($p = 0.0052$; Table 2). Based on two diagnostic criteria (clue cells and amine odor only), 81.3% of patients (165/204) in the ovule group were cured compared to 72.6% (130/180) of patients in the cream group ($p = 0.0173$). There was no significant difference between treatment groups regarding the distribution among Gram stain scores. The majority of patients had normal or intermediate scores at the first follow-up visit.

### Thirty-five-day assessment

By the second follow-up visit, there was no significant difference between treatment groups for the evaluable patient population, although cure rates were lower (59.7–61.2%) using three diagnostic criteria. Similarly, the cure rate was 78.9% in each group using two diagnostic criteria. The higher cure rate with two vs. three diagnostic criteria reflects the reduced stringency of the efficacy criteria and the influence of the pH criterion in lowering the defined cure rate.

No significant differences were noted between treatment groups regarding the distribution among

**Table 1** Patient demographics (intent to treat (ITT) patients)

| *Variable* | *CVO (n = 327)* | *CVO (n = 335)* | *Total (n = 662)* | *Treatment p value** |
|---|---|---|---|---|
| Age (years) | | | | 0.8961 |
| Mean ± SD | 29.5 ± 8.3 | 29.5 ± 8.0 | 29.5 ± 8.2 | |
| Range | 16–55 | 17–58 | 16–58 | |
| Weight (kg) (mean ± SD) | 69.0 ± 19.4 | 69.3 ± 17.5 | 69.2 ± 18.4 | 0.8286 |
| Range; number reporting | 40–172; *n* = 314 | 42–133; *n* = 329 | 40–172; *n* = 643 | |
| Race (*n*(%)) | | | | |
| White | 113 (34.6) | 112 (33.4) | 225 (34.0) | 0.8340 |
| Black | 148 (45.3) | 149 (44.5) | 297 (44.9) | |
| Oriental/Asian | 3 (0.9) | 2 (0.6) | 5 (0.8) | |
| Hispanic | 61 (18.7) | 68 (20.3) | 129 (19.5) | |
| Other | 2(0.6) | 4(1.2) | 6(0.9) | |

CVO, clindamycin vaginal ovule, 3-day treatment; CVC, clindamycin vaginal cream, 7-day treatment; *for age and weight, based on one-way analysis of variance (excluding 'not reported'); for race, based on two-tailed Fisher's exact test (based on white, black and all other races)

Gram stain score categories. The majority of patients had normal Gram stain scores (ovule group, 57.8%; cream group, 58.9%).

### ITT population

Overall clinical outcome was also similar between treatment groups when assessed in the ITT patient population (Table 3). In the ITT patient population, a significantly greater percentage of patients within the ovule group was cured at the first follow-up visit relative to the cream group using three diagnostic criteria (88.8% vs. 80.8%; $p = 0.0061$) and also when using two diagnostic criteria (84.3% vs. 77.3%; ($p = 0.0134$). Cure rates at the second visit ranged from 78.9% to 80.9%. A similar percentage of patients within each treatment group (79.1% of the reporting patients in the ovule group, 151/191; 83.7% of the reporting patients in the cream group, 128/153) rated their vaginal infections as having been cured.

### Safety

Safety was evaluated by patient-reported medical events. No statistically significant difference ($p = 0.740$) between the treatment groups was found in the percentage of patients reporting at least one medical event (33.3% ovule group, 109/327; 31.9% cream group, 107/335). The percentage of patients reporting medical events was similar between treatment groups, with the

**Table 2** Clinical cure rates and Gram stain results at 14- and 35-day assessments among evaluable patient population

| | *14-Day assessment* | | | *35-Day assessment* | | |
|---|---|---|---|---|---|---|
| | *Number (%) of patients* | | | *Number (%) of patients* | | |
| *Outcome* | *CVO* (n = *204)* | *CVC* (n = *180)* | p *value** | *CVO* (n = *204)* | *CVC* (n = *180)* | p *value** |
| Three diagnostic criteria | | | | | | |
| Cure | 175 (86.2) | 135 (75.4) | 0.0052 | 114 (59.7) | 93 (61.2) | 0.7781 |
| Failure | 28 (13.8) | 39 (21.8) | | 77 (40.3) | 59 (38.8) | |
| Two diagnostic criteria | | | | | | |
| Cure | 165 (81.3) | 130 (72.6) | 0.0173 | 150 (78.9) | 120 (78.9) | 1.0000 |
| Failure | 38 (18.7) | 44 (24.6) | | 40 (21.1) | 32 (21.1) | |
| Gram stain score | | | | | | |
| Normal (0–3) | 110 (55.8) | 74 (43.8) | 0.0672 | 108 (57.8) | 89 (58.9) | 0.9642 |
| Intermediate (4–6) | 63 (32.0) | 72 (42.6) | | 41 (21.9) | 33 (21.9) | |
| Compatible with BV (7–10) | 24 (12.2) | 23 (13.6) | | 38 (20.3) | 29 (19.2) | |

CVO, 3-day clindamycin vaginal ovule; CVC, 7-day clindamycin vaginal cream; *based on $\chi^2$; BV, bacterial vaginosis

**Table 3** Clinical outcome for ovule (CVO) and cream (CVC) groups using three and two diagnostic criteria for the intent to treat (ITT) population

| | *Three diagnostic criteria* | | | *Two diagnostic criteria* | | |
|---|---|---|---|---|---|---|
| | *Number (%) of patients* | | | *Number (%) of patients* | | |
| *Outcome* | *CVO* (n = *327)* | *CVC* (n = *335)* | p *value** *95% CI*** | *CVO* (n = *327)* | *CVC* (n = *335)* | p *value** *95% CI*** |
| Cure | 134 (56.3) | 113 (50.4) | 0.2072 | 164 (68.3) | 142 (62.3) | 0.1689 |
| Failure | 104 (43.7) | 111 (49.6) | −3.2, 14.9 | 76 (31.7) | 86 (37.7) | −2.6, 14.7 |
| Nonassessable | 89 | 111 | | 87 | 107 | |

CVO, 3-day clindamycin vaginal ovule; CVC, 7-day clindamycin vaginal cream; *based on $\chi^2$ test (three criteria, Breslow–Day test $p$ value = 0.1724) or Cochran–Mantel–Haenszel test (two criteria, Breslow–Day test $p$ value = 0.0665); **two-sided 95% CI for difference in cure rates between CVO and CVC groups exception of vaginal pain (ovule group, 3.4%; cream group, 0.9%), flu syndrome (ovule group, 0.9%; cream group, 2.7%), and headache (ovule group, 6.4%; cream group, 3.6%). Vaginal candidiasis was reported in five (1.5%) patients in the ovule group and three (0.9%) in the cream group. The vast majority of medical events reported in both treatment groups were of mild to moderate intensity (ovule group, 95.2%, 177/186; cream group, 87.1%, 149/171). Nine events in the ovule group (4.8%) and 19 events in the cream group (11.1%) were rated as severe (interfered with usual function). Fungal infections affecting either the skin or the urogenital system occurred in a similar percentage of patients in both treatment groups. The most frequently reported medical event was vulvovaginal itching (7.3% in the ovule group, 8.4% in the cream group). Very few patients discontinued medication because of medical events (ITT patients, 1 of 327 in the ovule group, 6 of 335 in the cream group).

### DISCUSSION

Therapeutic options for practitioners in the treatment of BV have increased from oral metronidazole to better tolerated and more preferred topical treatments, metronidazole gel and clindamycin cream. Comparative studies of these standard therapies have revealed no differences in efficacy or safety when used in the treatment of BV[6,10–13]. Topical regimens also offer the significant advantage of fewer unwanted systemic effects. Nonetheless, these treatments are not without inconveniences, and patient compliance and preference are an important consideration for the treatment of BV in the general population. The search, therefore, continues for faster, more convenient alternatives for the treatment of BV. The vaginal ovule represents a more convenient, less messy alternative, and women may prefer this form of dosing to conventional cream applicators. Moreover, the shortened 3-day regimen and once-per-day dosing will likely facilitate improved patient compliance and adherence to therapy.

This study demonstrates that a 3-day course of clindamycin vaginal ovules (100-mg ovule, administered intravaginally at bedtime for 3 consecutive days) is as effective as a 7-day course of clindamycin vaginal cream (5 g (1 applicator equals 100 mg clindamycin) administered intravaginally at bedtime for 7 consecutive days) in the treatment of symptomatic BV. Assessment of cure rates using either two diagnostic criteria (clue cells and amine odor) or three diagnostic criteria (clue cells, amine odor and pH) consistently demonstrated no significant difference between the two treatment regimens. The influence of the pH criterion in lowering the observed cure rate is noteworthy and suggests that pH may be a less reliable diagnostic marker of cure. Nonetheless, inclusion of the pH criterion had no effect on the observed similarity in efficacy of the two treatment regimens.

No significant difference in the safety of these two alternative clindamycin dosing regimens was observed in the current study. Incidence of medical events was similar for both treatment groups, and most events were of mild to moderate intensity. Incidence of fungal infections and vulvovaginal disorder (mainly vulval and vaginal itching) was also similar between treatment groups. Very few patients discontinued medication because of medical events. No significant safety problem with the clindamycin vaginal ovule was observed. Short-term therapy with clindamycin vaginal ovules as demonstrated in this study is therefore effective and well-tolerated and offers women a shortened, more convenient dosing alternative for the treatment of BV.

### REFERENCES

1. Sobel JD. Vaginitis. *N Engl J Med* 1997;337: 1896–1903
2. Hillier S, Holmes KK. Bacterial vaginosis. In Holmes KK, Sparling PF, Mardh PA, *et al*., eds. *Sexually Transmitted Diseases*, 3rd edn. New York: McGraw Hill, 1999:563–86
3. Hay PE. Therapy of bacterial vaginosis. *J Antimicrob Chemother* 1998;41:6–9
4. Gravett MG, Hummel D, Eschenbach DA, Holmes KK. Preterm labor associated with subclinical amniotic fluid infection and with bacterial vaginosis. *Obstet Gynecol* 1986;67:229–37

5. Lamont RF, Taylor-Robinson D, Newman M, *et al.* Spontaneous early preterm labor associated with abnormal genital tract bacterial colonization. *Br J Obstet Gynaecol* 1986;93:804–10
6. Drug and Therapeutics Bulletin 1998. Management of bacterial vaginosis. *Drug Ther Bull* 1998; 36:33–5
7. Peipert JF, Montagno A, Cooper AS, Sung J. Bacterial vaginosis as a risk factor for upper genital tract infection. *Am J Obstet Gynecol* 1997;177: 1184–7
8. Joesoef MR, Schmid GP, Hillier SL. Bacterial vaginosis: review of treatment options and potential clinical indications for therapy. *Clin Infect Dis* 1999;28(Suppl 1):S57–S65
9. Mikamo H, Kawazoe K, Izumi K, *et at.* Comparative study on vaginal or oral treatment of bacterial vaginosis. *Chemotherapy* 1997;43:60–8
10. Ferris D, Litaker MS, Woodward L, *et al.* A comparison of oral metronidazole, metronidazole vaginal gel, and clindamycin vaginal cream. *J Fam Pract* 1995;41:443–9
11. Schmitt C, Sobel JD, Meriwether C. Bacterial vaginosis: treatment with clindamycin cream versus oral metronidazole. *Obstet Gynecol* 1992; 79:1020–30
12. Andres FJ, Parker R, Hosein I, Benrubi GI. Clindamycin vaginal cream versus oral metronidazole in the treatment of bacterial vaginosis: a prospective double-blind clinical trial. *South Med J* 1992;85:977–1080
13. Fischbach F, Petersen EE, Weissenbacher ER, *et al.* Efficacy of clindamycin vaginal cream versus oral metronidazole in the treatment of bacterial vaginosis. *Obstet Gynecol* 1993;82:405–10
14. Powley GW, Timm JA, Greenwald CA. Comparison of two dosing regimens of 2% clindamycin vaginal cream for the treatment of bacterial vaginosis – 3 days vs. 7 days (protocol M/1115/0020). Upjohn Technical Report 9156-93-006, December 30, 1993
15. Dhar J, Arya OP, Timmins DJ, *et al.* Treatment of bacterial vaginosis with a three-day course of clindamycin vaginal cream: a pilot study. *Genitourin Med* 1994;70:121–3
16. Ahmed-Jushuf IH, Shahmanesh M, Arya OP. The treatment of bacterial vaginosis with a three-day course of 2% clindamycin cream: results of a multicenter, double-blind, placebo-controlled trial. *Genitourin Med* 1995;71:254–6
17. Amsel R, Totten PA, Spiegel CA, *et al.* Nonspecific vaginitis. Diagnostic criteria and microbial and epidemiologic associations. *Am J Med* 1983; 74:14–22
18. Nugent RP, Krohn MA, Hillier SL. Reliability of diagnosing bacterial vaginosis is improved by a standardized method of Gram stain interpretation. *J Clin Microbiol* 1991;29:297–301

RECEIVED 12/01/99; ACCEPTED 11/30/00
